# Long-Term Survival of Patients with Stage T1N0M1 Renal Cell Carcinoma

**DOI:** 10.3390/cancers15245715

**Published:** 2023-12-06

**Authors:** Viktoria Schütz, Huan Lin, Adam Kaczorowski, Stefanie Zschäbitz, Dirk Jäger, Albrecht Stenzinger, Anette Duensing, Jürgen Debus, Markus Hohenfellner, Stefan Duensing

**Affiliations:** 1 Department of Urology, University Hospital Heidelberg, Im Neuenheimer Feld 420, 69120 Heidelberg, Germany; 2 Molecular Urooncology, Department of Urology, University Hospital Heidelberg, Im Neuenheimer Feld 517, 69120 Heidelberg, Germany; 3 Department of Medical Oncology, National Center for Tumor Diseases Heidelberg, University Hospital Heidelberg, Im Neuenheimer Feld 460, 69120 Heidelberg, Germany; 4 Institute of Pathology, University Hospital Heidelberg, Im Neuenheimer Feld 224, 69120 Heidelberg, Germany; 5 Precision Oncology of Urological Malignancies, Department of Urology, University Hospital Heidelberg, Im Neuenheimer Feld 517, 69120 Heidelberg, Germany; 6 Department of Radiation Oncology, University Hospital Heidelberg, Im Neuenheimer Feld 400, 69120 Heidelberg, Germany

**Keywords:** renal cell carcinoma, metastatic renal cell carcinoma, T1N0M1, long-term survival

## Abstract

**Simple Summary:**

Localized renal cell carcinoma (RCC) has an excellent prognosis. However, once metastatic, patient prognosis declines significantly. There is a rare patient subgroup with localized RCC stage T1 with distant metastasis (M1) and no lymph node involvement (N0). In this study, the survival of patients diagnosed with clear cell RCC (ccRCC) stage T1N0M1 were evaluated in comparison to ccRCC patients stage T1 without metastases (N0M0). As expected, stage T1N0M1 ccRCC patients showed a significantly worse outcome than stage T1N0M0 patients. To further characterize tumor characteristics of stage T1N0M1 ccRCC patients, CD8+ tumor infiltrating lymphocytes (TILs) were analyzed, as it is known that a number of CD8+ TILs is associated with a worse prognosis. It could be shown that tumor specimens from stage T1N0M1 ccRCC patients harbor a substantially higher number of CD8+ TILs than specimens from stage T1N0M0 ccRCC patients, thus resembling advanced stage ccRCC. Nevertheless, long-term survival of stage T1N0M1 ccRCC patients is possible.

**Abstract:**

Metastatic renal cell carcinoma (RCC) is among the most lethal urological malignancies. However, small, localized RCCs (≤7 cm, stage T1) have an excellent prognosis. There is a rare patient subgroup diagnosed with synchronous distant metastasis (T1N0M1), of which very little is known in terms of survival outcomes and underlying disease biology. Herein, we examined the long-term survival of 27 patients with clear cell RCC (ccRCC) stage T1N0M1 in comparison to 18 patients without metastases (T1N0M0). Tumor tissue was stained by immunohistochemistry for CD8+ tumor infiltrating lymphocytes (TILs). As expected, patients with stage T1N0M1 showed a significantly worse median cancer specific survival (CSS; 2.8 years) than patients with stage T1N0M0 (17.7 years; HR 0.077; 95% CI, 0.022–0.262). However, eight patients (29.6%) with ccRCC stage T1N0M1 survived over five years, and three of those patients (11.1%) survived over a decade. Some of these patients benefitted from an intensified, multimodal treatment including metastasis-directed therapy. The number of CD8+ TILs was substantially higher in stage T1N0M1 ccRCCs than in stage T1N0M0 ccRCCs, suggesting a more aggressive tumor biology. In conclusion, long-term survival is possible in patients with ccRCC stage T1N0M1, with some patients benefitting from an intensified, multimodal treatment approach.

## 1. Introduction

Once metastatic, renal cell carcinoma (RCC) has a poor prognosis with a 5-year cancer specific survival (CSS) rate of 20% or less [1]. In contrast, the prognosis is excellent for patients with stage T1 non-metastatic tumors with a 5- and 10-year CSS of approximately 90% [2,3], with most RCC-related deaths occurring within the first 5 years after diagnosis [3]. However, there is a rare subgroup of RCC patients with synchronous distant metastases at time of diagnosis (stage T1N0M1). Very little is known about the tumor biology and long-term prognosis of these patients. It is known, however, that the rate of synchronous metastasis in T1 RCC is dependent on the histological subtype, grading, sarcomatoid differentiation, and tumor size [4]. For clear cell RCC (ccRCC), the rate of synchronous metastasis in T1 disease ranges from 0.2 to 8.9% depending on tumor grade [4]. The number of distant metastases has recently been suggested as a prognostic factor, with patients presenting with more than three metastases having a worse overall survival (OS) compared to patients with three or fewer metastases [5].

It has previously been shown that advanced ccRCC is characterized by a high degree of genomic intratumoral heterogeneity (ITH) [6]. In addition, ccRCC also shows a high degree of functional ITH, i.e., the formation of spatial niches that are populated by tumor cells with certain characteristics such as a high proliferative activity [7,8]. However, results from our group show that the extent of functional ITH does not correlate with tumor stage [7]. Moreover, there were no mutational events that could explain functional ITH and niche formation. In contrast, there is evidence that the tumor microenvironment may play a role in shaping ITH in ccRCC [9]. The tumor microenvironment has been proposed to play an important role in carcinogenesis and tumor progression [10,11,12]. A key component of the tumor microenvironment in ccRCC is the immune microenvironment. T cells are the dominant immune cell type in the tumor immune microenvironment in ccRCC [13]. In contrast to other tumor entities, a high infiltration with CD8+ T cells is associated with a worse patient survival [14,15,16,17,18,19]. This is likely due to the fact that most CD8+ cytotoxic T lymphocytes are dysfunctional or terminally exhausted [11,20,21]. Therefore, reactivation of cytotoxic T cells through immune checkpoint blockade, together with anti-angiogenetic agents, plays a crucial role in the systemic treatment of patients with advanced RCC [22,23,24]. Although ccRCCs stage T1N0M0 were not distinguishable from ccRCCs stage T1N0M1 based on the analysis of functional ITH [7], it is conceivable that these two patient subgroups show differences in terms of prognosis. 

In the current study, we analyzed long-term survival, the CD8+ T cell immune landscape, as well as the impact of a multimodal treatment in patients presenting with ccRCC stage T1N0M1.

## 2. Patients and Methods

### 2.1. Patients

Forty-five patients with T1 ccRCC were included in this analysis. All patients received a radical or partial nephrectomy between 1990 and 2005. Maximum follow-up time was 27 years for patients with stage T1N0M0 (n = 18) and 21 years for patients with stage T1N0M1 (n = 27). There was no evidence of nodal involvement in any patient (c/pN0). Information was retrieved from the clinical information system of the University Hospital Heidelberg, as well as the tumor database of the Department of Urology of the University Hospital Heidelberg. Patients gave written informed consent for the use of their data and tissue for research and publication. Tissue samples were provided by the tissue bank of the National Center for Tumor Diseases (NCT) Heidelberg, Germany, in accordance with the regulations of the tissue bank and the approval of the ethics committee of the Medical Faculty Heidelberg of the University of Heidelberg. 

### 2.2. Immunohistochemistry

Tumor tissue was analyzed for CD8+ tumor infiltrating lymphocytes (TILs) using immunohistochemistry (IHC). As a control, tissue samples from ccRCCs stage T3/T4 were used (n = 5). These patients were not included in the outcome analysis. 

Formalin-fixed, paraffin-embedded tissue sections were first baked at 37 °C overnight. On day one, the slides were deparaffinized by immersion in xylol for a total of 12 min. The slides were then rehydrated in a graded series of ethanol. After washing the slides in distilled water, antigen retrieval was performed using a steamer and target retrieval solution (Dako, Glostrup, Denmark) for 35 min. After cooling down, the slides were washed in distilled water and phosphate-buffered saline (PBS), twice each. Peroxidase quenching was performed using 3% H_2_O_2_ in methanol for 10 min. To block non-specific antibody binding, 10% normal goat serum (Dako) was applied for 30 min. Slides were then incubated with the primary antibody in a humidified chamber at 4 °C overnight. A polyclonal anti-CD8 alpha antibody was used (Abcam, Cambridge, UK; ab4055, 1:50 in PBS). On day two, the slides were washed in PBS for 30 min and afterwards incubated with a secondary biotin-conjugated antibody (goat anti-rabbit IgG H&L, Abcam, ab97049, 1:200) at 37 °C for three hours. After washing the slides in PBS they were incubated with a Streptavidin-POD-Conjugate (Roche Diagnostics, Indianapolis, IN, USA) diluted in PBS (1:1250) at room temperature for 30 min. Slides were then stained with DAB solution (DAB Substrate Kit, Abcam, ab64238) and transferred into tap water. Counterstaining was performed with hematoxylin for 5 s (Hematoxylin Solution, Gill No.1, Sigma-Aldrich, St. Louis, MO, USA). After washing in PBS, the tissue was dehydrated in an ethanol series (50, 70, 90 and 100%), washed again with xylol, and mounted using Histomount (Invitrogen, Waltham, MA, USA). 

CD8+ TILs were counted using a Leica DM750 microscope (Leica, Wetzlar, Germany) equipped with a 20× objective and a 10× eyepiece leading to a field of view of 0.785 mm^2^. CD8+ TILs were counted separately for the tumor center and tumor periphery. Results were documented using a Leica DM5000 B microscope equipped with a K3C camera (Leica). 

### 2.3. Statistical Analysis

*p*-values were calculated using the Mann–Whitney U test or Fisher’s exact test. Statistical significance was set at *p* < 0.05. The Kaplan–Meier method was used to calculate CSS and OS with log-rank statistics. Descriptive analysis was completed using Microsoft^®^ Excel^®^. Statistical analysis was conducted with IBM^®^ SPSS Statistics Version 27. 

## 3. Results

### 3.1. Long-Term Survival of Patients with ccRCC Stage T1N0M1

A total of 45 ccRCC patients were included in this retrospective, single-center analysis. Of these, 27 patients presented with stage T1N0M1 and 18 patients presented with stage T1N0M0 at the time of diagnosis (Table 1). Patient characteristics were well balanced with respect to age, gender, and T stage. Significant differences were noted in death from ccRCC (*p* < 0.001) and death from any cause (*p* < 0.001), as shown in Table 1. 

The number of deaths from any cause was 33 (73.3%) in the entire cohort (n = 45), eight (44.4%) in the T1N0M0 group (n = 18), and 25 (92.6%) in the T1N0M1 group (n = 27). The number of deaths from RCC was 26 (57.8%) in the entire cohort, three (16.7%) in the T1N0M0 group, and 23 (85.2%) in the T1N0M1 group.

**Figure 1:**
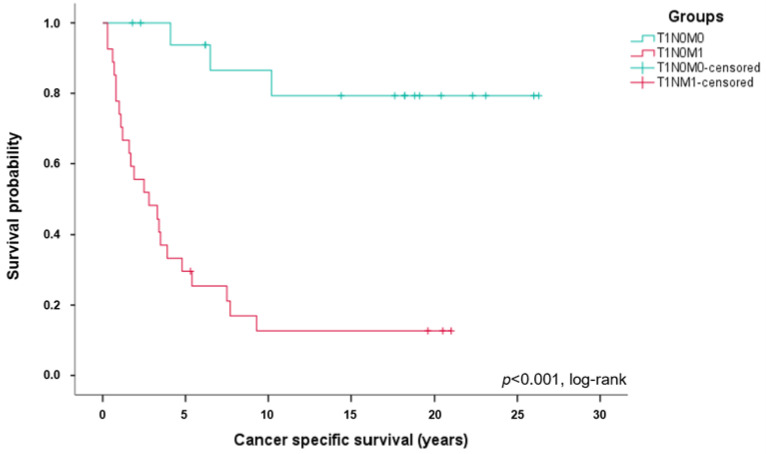
Significantly worse cancer-specific survival in T1N0M1 ccRCC patients. Kaplan–Meier curves showing cancer specific survival in stage T1N0M1 (n = 27, red) and T1N0M0 (n = 18, green) ccRCC patients.

Long-term survival differed significantly between ccRCC patients with stage T1N0M1 and T1N0M0 tumors. A Kaplan–Meier survival analysis (Figure 1) revealed a median CSS of patients with stage T1N0M1 of 2.8 years compared to 17.9 years in patients with T1N0M1 (*p* < 0.001, log-rank; HR, 0.077; 95% CI, 0.022–0.262). A total of 23 patients with stage T1N0M1 (85.2%) died from ccRCC, but only three ccRCC patients with stage T1N0M0 (16.7%) died from the tumor (*p* < 0.001). 

**Figure 2:**
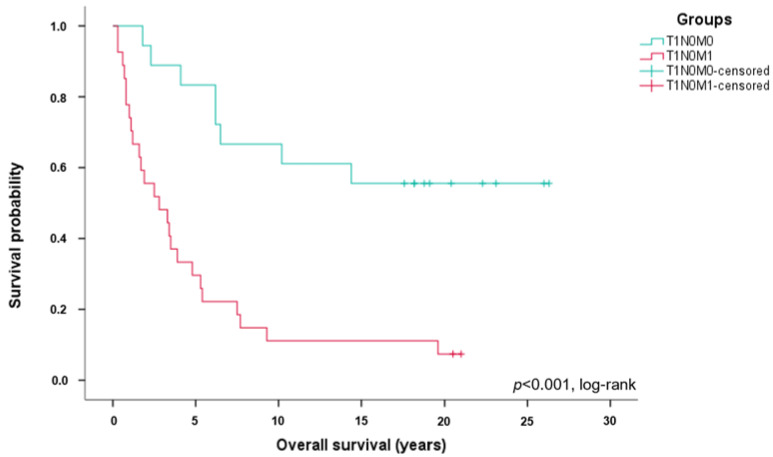
Significantly worse overall survival in T1N0M1 ccRCC patients. Kaplan–Meier curves showing overall survival in stage T1N0M1 (n = 27, red) and T1N0M0 (n = 18, green) ccRCC patients.

The 5-year survival rate in the T1N0M1 group was 29.6% (n = 8) compared to 83.3% (n = 15) in the T1N0M0 group. The 10-year survival rate of ccRCC patients with stage T1N0M1 was 11.1% (n = 3) compared to 66.7% (n = 12) in T1N0M0 patients. The OS was also significantly worse in patients with ccRCC stage T1N0M1 compared to patients with stage T1N0M0 (*p* < 0.001, Figure 2).

### 3.2. Patients with ccRCC Stage T1N0M1 Benefit from an Intensified, Multimodal Therapy

Twenty-four patients with stage T1N0M1 (88.9%) underwent further treatment for ccRCC, while only five patients with stage T1N0M0 (27.8%) needed further oncological treatment (*p* < 0.001). Subsequent treatment modalities included surgical resection of metastases, radiotherapy, tumor embolization, as well as systemic treatment including immunotherapy, chemotherapy, and treatment with mTOR- or tyrosine kinase inhibitors (TKIs; Table 2). 

**Figure 3:**
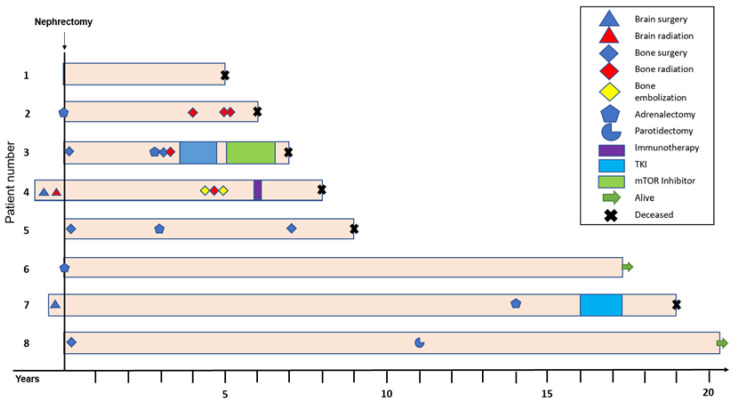
Long-term survival of T1N0M1 ccRCC patients. Swimmer plot showing eight T1N0M1 ccRCC patients with cancer-specific survival of ≥5 years out of a total of 27 (29.6%) patients. The plot illustrates the different treatment modalities the patients received over time, including surgical resection of metastases, radiotherapy, as well as systemic treatment with TKIs, mTOR inhibitors, or immunotherapy. Three patients (11.1%) survived for ≥10 years.

In order to better understand the role of multimodal treatment in patients with ccRCC stage T1N0M1, the oncological therapies were analyzed in patients surviving five years or longer (n = 8, 29.6%; Figure 3).

There was considerable clinical heterogeneity in these patients. While most patients required additional treatment including different treatment modalities (Figure 3), one patient (patient #6) received a tumor nephrectomy with simultaneous adrenalectomy and no additional oncological treatment. Three patients (11.1%) survived for 15 years or longer. 

### 3.3. Enhanced CD8+ T Cell Infiltration in T1N0M1 ccRCC

**Figure 4:**
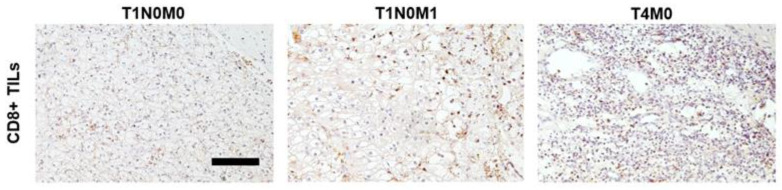
CD8+ T cell infiltration in ccRCC patients. Representative photomicrographs of CD8+ TILs in patients diagnosed with ccRCC stage T1N0M0, T1N0M1, or T4M0 (as a comparison). Tissues were stained for CD8 by immunohistochemistry. Scale bar = 250 µm.

In contrast to other malignancies, a high infiltration with CD8+ T cells is associated with a poor prognosis in RCC [15]. In order to assess whether patients with T1N0M1 ccRCC differ in their CD8+ T cell infiltrate, IHC was performed and results were compared to patients with ccRCC stage T1N0M0 and advanced stage ccRCC (T3/T4, n = 5). CD8+ TILs were counted in the tumor periphery and tumor center (Figure 4). 

**Figure 5:**
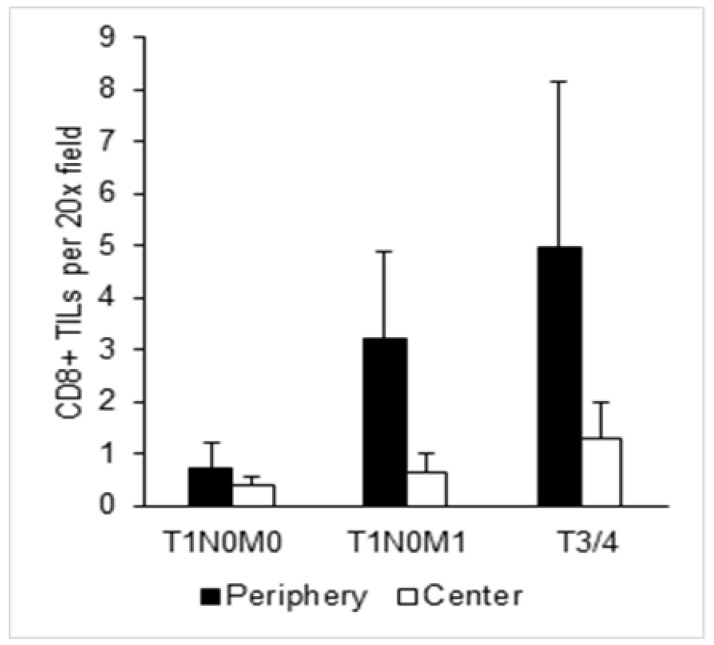
CD8+ T cell infiltration in ccRCC patients. Quantification of CD8+ TILs per 20× microscopic field in ccRCC patients stage T1N0M0, T1N0M1, or T3/4. The tumor center and tumor periphery were analyzed separately. Bar graphs indicate mean and standard error of at least five fields counted per region.

Although not reaching statistical significance, our results show that the number of CD8+ TILs is considerably higher in the tumor periphery in ccRCC stage T1N0M1 when compared to ccRCC stage T1N0M0 and hence resemble advanced stage ccRCC (Figure 5).

## 4. Discussion

Patients presenting with RCC stage T1 have an excellent prognosis. However, once metastatic, the patient’s prognosis worsens significantly. On rare occasions, patients with RCC stage T1 are diagnosed with a synchronous metastatic spread. Almost nothing is known about the survival outcomes of these patients and the underlying disease biology. In this retrospective, single-center analysis, a total of 27 ccRCC patients presenting with stage T1N0M1 disease diagnosed between 1990 and 2005 were included, in comparison to 18 patients with stage T1N0M0 disease. As expected, patients presenting with ccRCC stage T1N0M1 showed a significantly worse CSS and OS when compared to ccRCC patients with stage T1N0M0. Remarkably, however, a subgroup of patients with ccRCC stage T1N0M1 survived longer than five or even 10 years. Further analysis revealed that a subset of these patients had benefitted from an intensified, multimodal treatment. When we analyzed the immune landscape of the primary tumors, we detected higher numbers of CD8+ TILs in the tumor periphery of stage T1N0M1 ccRCCs than stage T1N0M0 ccRCCs as a possible indication for a more aggressive tumor behavior. 

Patients with RCC stage T1N0M1 represent a rare subgroup of RCC patients. In RCC patients stage T1, synchronous metastasis occurs only in up to approximately 10% [25]. A correlation between tumor size and risk for synchronous metastasis has previously been reported [25,26]. 

The median CSS observed in our T1N0M1 cohort was 2.8 years, which is significantly better than the median CSS in patients with metastatic RCC in general. The SEER database reported a median of 0.8 years for patients with M1 disease in the era of TKI treatment (2007–2013) and 0.7 years before the introduction of TKIs (2000–2006) [27]. Our finding likely reflects the effect of the local T stage, suggesting that smaller primary tumors are less prone to metastatic dissemination and tumor progression [25,26,28]. 

The 5- and 10-year survival rates found in our T1N0M1 cohort were 29.6% and 11.1%, respectively. While these survival rates were considerably lower than in ccRCC patients with stage T1N0M0 disease, they are higher than what has been reported in RCC patients with M1 disease in general, which had a 5-year survival rate of approximately 10% [29]. Given these relatively high survival rates, we performed a further analysis of these patients and detected a considerable clinical heterogeneity. Whereas some patients did not require additional treatment after the initial surgery, others clearly benefitted from an intensified, multimodal therapy. The long-term survival is also remarkable with respect to the fact that immune checkpoint inhibitors were not available at the time of treatment. 

In a previous study from our group [7], of which the current study is a follow-up, no differences in the level of intratumoral heterogeneity in patients with stage T1N0M1 and patients with stage T1N0M0 were detected [7]. However, the previous study did not take the tumor microenvironment into account. In the present analysis, we show that there is a difference in the immune landscape, with T1N0M1 tumors clearly showing a higher infiltration with CD8+ T cells compared to T1N0M0 tumors. In contrast to other malignancies, a high infiltration with CD8+ T cells is associated with an unfavorable prognosis in RCC [14,15,16,17,18,19]. This is most likely due to the fact that many of the CD8+ cytotoxic T cells are dysfunctional or terminally exhausted [11]. Therefore, our finding suggests that the primary tumors of patients with stage T1N0M1 disease may be characterized by a higher degree of genomic instability and neo-antigen expression [6,30]. 

A limitation of this analysis is the small sample size. As a consequence, a uni- or multivariate Cox regression analysis could not be conducted. 

## 5. Conclusions

In conclusion, the present study highlights that an intensified, multimodal treatment can be beneficial in stage T1N0M1 ccRCC patients, leading to a more favorable CSS and OS compared to RCC patients with metastatic disease in general. Further studies on the immunological and genomic aspects of ccRCC stage T1N0M1 are clearly warranted. 

## Figures and Tables

**Table 1:** Clinico-pathological patient characteristics.

Characteristics	T1N0M0	T1N0M1	All	*p*-Value
Patients, n (%)	18 (40)	27 (60)	45 (100)	
Age at time of surgery, years				0.098
Median	54.5	62	60	
IQR	48.5–66.25	56–64	52.5–65	
Gender, n (%)				0.188
Male	10 (55.6)	21 (77.8)	31 (68.9)	
Female	8 (44.4)	6 (22.2)	14 (31.1)	
c/pTNM initial, n (%)				
T stage				
pT1a	11 (61.1)	13 (48.1)	24 (53.3)	0.543
pT1b	7 (38.9)	14 (51.9)	21 (46.7)	
N stage				
c/pN0	18 (100)	27 (100)	45 (100)	n/a
cN1	0	0	0	
M stage				
M0	18 (100)	0	18 (40)	<0.001
M1	0	27 (100)	27 (60)	
Tumor size, cm				0.709
Median	3.75	4.5	4.0	
IQR	2.9–6.0	3.0–6.0	3.0–6.0	
Site of metastasis, n (%) at time of diagnosis	n/a			
Bone		8 (29.6)		
Brain		2 (7.4)		
Adrenal gland		3 (11.1)		
Lung		3 (11.1)		
Multiple sites		10 (37.0)		
Missing information		1 (3.7)		
Number of metastases at time of diagnosis, n (%)	n/a			
≤3		17 (63.0)		
>3		2 (7.4)		
Missing information		8 (29.6)		
Confirmation of M1, n (%)	n/a			
Histology		20 (74.1)		
Imaging		2 (7.4)		
Missing information		5 (18.5)		
Histology primary tumor, n (%)				0.228
Clear cell	18 (100)	27 (100)	45 (100)	
Death from RCC, n (%)				<0.001
Yes	3 (16.7)	23 (85.2)	26 (57.8)	
No	5 (27.8)	1 (3.7)	6 (13.3)	
Unknown	0	1 (3.7)	1 (1.2)	
Death from any cause, n (%)				<0.001
Yes	8 (44.4)	25 (92.6)	33 (73.3)	
No	10 (55.6)	2 (7.4)	12 (26.7)	
Follow-up time, years				<0.001
Median	17.9	2.8	5.3	
IQR	6.2–20.87	1.0–5.4	1.75–18.2	

**Table 2:** Treatment modalities.

Characteristics	T1N0M0	T1N0M1	All
Patients, n (%)	18 (40)	27 (60)	45 (100)
Primary surgery, n (%)			
Radical nephrectomy	10 (55.6)	27 (100)	37 (82.2)
Partial nephrectomy	8 (44.4)	0	8 (17.8)
Further treatment, n (%)			
Surgery	4 (22.2)	17 (63.0)	21 (46.7)
Radiotherapy	3 (16.7)	20 (74.1)	23 (51.1)
Tumor embolization	0	1 (3.7)	1 (2.2)
Immunotherapy	0	13 (48.1)	13 (28.9)
TKI	2 (11.1)	3 (11.1)	5 (11.1)
mTOR Inhibitors	1 (5.6)	2 (7.4)	3 (6.7)
Chemotherapy	0	2 (7.4)	2 (4.4)
No. of subsequent treatments, n (%)			
0	13 (72.2)	3 (11.1)	16 (35.6)
1	2 (11.1)	3 (11.1)	5 (11.1)
2	0	3 (11.1)	3 (6.7)
3	1 (5.6)	8 (29.6)	9 (20)
≥4	2 (11.1)	10 (37.0)	12 (26.7)

## Data Availability

The data presented in this study are available within the article.
